# Co‐designing for quality: Creating a user‐driven tool to improve quality in youth mental health services

**DOI:** 10.1111/hex.12694

**Published:** 2018-04-29

**Authors:** Christina L. Hackett, Gillian Mulvale, Ashleigh Miatello

**Affiliations:** ^1^ Health Policy McMaster University Hamilton ON Canada; ^2^ DeGroote School of Business McMaster University Hamilton ON Canada

**Keywords:** caregivers, child and youth mental health services, experience‐based co‐design, quality improvement, service providers, youth

## Abstract

**Background:**

Although high quality mental health care for children and youth is a goal of many health systems, little is known about the dimensions of quality mental health care from users’ perspectives. We engaged young people, caregivers and service providers to share experiences, which shed light on quality dimensions for youth mental health care.

**Methods:**

Using experience‐based co‐design, we collected qualitative data from young people aged 16‐24 with a mental disorder (n = 19), identified caregivers (n = 12) and service providers (n = 14) about their experiences with respect to youth mental health services. Experience data were collected using multiple approaches including interviews, a suite of online and smartphone applications (n = 22), and a co‐design event (n = 16) and analysed to extract touch points. These touch points were used to prioritize and co‐design a user‐driven prototype of a questionnaire to provide feedback to service providers.

**Findings:**

Young people, caregiver and service provider reports of service experiences were used to identify aspects of care quality at eight mental health service contact points: Access to mental health care; Transfer to/from hospital; Intake into hospital; Services provided; Assessment and treatment; Treatment environment; and Caregiver involvement in care. In some cases, low quality care was harmful to users and their caregivers. Young people co‐designed a prototype of a user‐driven feedback questionnaire to improve quality of service experiences that was supported by service providers and caregivers at the co‐design event.

**Conclusion:**

By using EBCD to capture in‐depth data regarding experiences of young people, their caregivers and service providers, study participants have begun to establish a baseline for acceptable quality of mental health care for young people.

## 1 INTRODUCTION

Incorporating patient experience into quality improvement practices is a stated policy goal in many jurisdictions,^1^, ^2^ and having a patient‐centred health service is an identified organizational goal across various levels of health system governance.^1^, ^3^ Perspectives and experiences of individuals accessing health care are valuable in creating and monitoring definitions of quality health services, as well as improving clinical safety of health services and health outcomes.^4^, ^5^ However, methodological and practical challenges to incorporating youth perspectives for quality improvement can exist in child and youth mental health services (CYMHS). As with other hard‐to‐reach groups in society, young people may experience a power imbalance when asked to provide feedback, and prior negative experiences accessing mental health care may make them unwilling to engage in participatory quality improvement efforts.^6^, ^7^ Nonetheless, young people have been engaged to create quality indicators for service engagement^8^ as well as quality indicators for mental health in the primary care setting.^9^ Across CYMHS more broadly, the fluidity with which young people access services across multiple organizations within and outside of health care makes it difficult to define quality dimensions and indicators that pertain to the networks comprising the “system” and operationalize system‐wide quality improvement efforts. A further challenge is that the perspectives and experiences of caregivers and service providers in CYMHS systems improvement are typically not included in more than a tokenistic way, if at all.^10^

Experience‐based co‐design (EBCD) has been highlighted as a best‐practice approach to engaging users in quality improvement in mental health care.^11^ Using an experience‐based approach in research can engage young people with mental health issues, reduce stigma and empower them through service co‐design^12^ to share their specific needs and approaches to seeking and receiving mental health care.^13^ The EBCD approach also incorporates the experiences of caregivers and service providers who work together with youth to co‐design quality improvements that reflect all three perspectives.

In this paper, we sought to understand dimensions of quality in CYMHS based on qualitative data that was gathered as part of a larger EBCD study (the *my*Co‐Design study)^14^ focusing on the coordination of mental health services for young people aged 16‐24, across multiple settings in the south‐western region of the province of Ontario, Canada. Ontario was selected because it is Canada's most populous province and has set a high priority on improving the quality of mental health services for this age group.^15^, ^16^ For example, in 2011, Ontario's Comprehensive Mental Health and Addictions Strategy was released, with the first 3 years focusing on the mental health of children and youth,^17^ and more recently, a number of national^18^, ^19^ and provincial efforts are underway to improve mental health system coordination and quality of care for this age group.^20^ When it comes to service coordination, a number of structural challenges for this age group exist in the Ontario context. Despite efforts to improve local coordination for young people accessing CYMHS, jurisdictional issues continue to complicate and impede transitions between child and adult mental health services, as well as coordination across agencies, regions and systems^1^ .

The objectives of this paper are threefold: (i) to present the process and outcomes of EBCD and showcase how experience data can inform the development of quality indicators for CYMHS across the inherent complexity of the multiple services and settings involved; (ii) to understand which quality factors hold most promise in improving experiences of mental health services; and (iii) to present a prototype of a tool co‐designed by youth, caregivers (family member or other lay person identified by the youth) and service providers in Ontario that can provide user feedback to providers with the objective of improving the quality of service experiences in CYMHS. We place particular emphasis on the experiences of young people as service users and how these can be a basis for considering the highs and lows in service experiences as potential indicators of quality. We also focus on a prototype driven by youth and supported by their identified caregivers and service providers, as a tool that can enhance communication between youth and service providers, and ultimately improve the experiences of youth mental health services.

## 2 METHODS

### 2.1 Overarching research design

Experience‐based co‐design (EBCD) is an approach informed by the design sciences, with evidence‐informed applications in health care.^10^ EBCD employs multiple perspectives (typically service users, an identified caregiver‐family member or other support person, and a service provider), to diagnose, intervene, implement and evaluate health services with the ultimate goal of co‐designing service improvements based on experiences.^21^ Participants’ experiences are gathered and analysed for “touch points” that represent significant experiences eliciting emotive responses in their health service use trajectory. The strong emotional response suggests the experience has touched upon a core value with respect to health care that may be a signal of health‐care quality.^22^ In addition to traditional qualitative interviews, participants in the *my*Co‐Design study were asked to use the suite of apps (*my*Exp apps) consisting of a mobile app (young people) and a web‐based app (caregivers and service providers) developed to ask users to provide feedback on youth mental health service experiences from their own perspectives in real‐time. The *my*EXP apps capture experiences when prompted following each appointment, or when spontaneously entered throughout the study period^2^ .

Once touch points are identified for each perspective (youth, caregiver and service provider), they are categorized as highs, or positive experiences of care, and lows or negative experiences of care. The touch points are mapped according to various stages in the journey through the mental health‐care system. Typically maps are validated at focus groups by participant type, where each group can provide feedback and member‐check the research team's findings (see Mulvale et al.^23^ for a more detailed explanation). A key stage in the EBCD process is an event during which the three participant types are brought together to co‐design solution prototypes for issues at key touch points. Subsequent EBCD phases can include further development, implementation and evaluation of the prototype or solution, within relevant systems/organizations.

These various stages of the EBCD process correspond to what has been described as the “Double Diamond” of design,^24^ where there is first an expansive process to explore all dimensions of the problem (discover), followed by a narrowing process to decide what to focus on (define), which comprises the first diamond. The second diamond begins from the narrowed focus of the problem to generate a wide range of potential solutions (develop), followed by a narrowing to a small number of solutions that have the potential to work from all perspectives (deliver). In the EBCD process applied here, the app data and interviews generated large numbers of touch points in understanding the problem. The focus group activities narrowed these through experience mapping by perspective. The research team subsequently synthesized these to three overarching touch points corresponding to each participant perspective, which became the foci for the co‐design event. At the co‐design event, participants from each perspective first individually and collectively brainstormed solutions. We adopted an inverted stoplight technique^25^ for the brainstorming process: wherein green light meant “go, go, go” as an individual brainstorming technique, yellow light meant slow down and cluster suggestions across group members, and red light meant stop and collectively assess feasibility select among and combine features of the group's suggested solutions. Each group then collectively drew a visual representation of their solution prototype. Participants from the other groups next circulated and commented on each other's prototypes to enhance them. Notes were taken that reflected these commentaries to be incorporated in the final prototypes that reflected all participant perspectives.

### 2.2 Data collection

Data from the *my*Co‐Design study were collected between August 2015 and July 2017 and used to identify touch points to understand what is driving high versus low quality mental health care from the perspectives of youth, caregivers and service providers and to capture their suggestions for prototype solutions for quality improvement (see Table 1). Three data sources were used as follows: (i) semi‐structured interviews conducted with young people aged 16 to 24 (n = 19), caregivers (n = 12) and service providers (n = 14); (ii) qualitative data from the *my*Exp app questionnaires (12 youth, 6 caregivers, 4 service providers); and (iii) notes taken on prototypes developed at the co‐design event^3^ . The co‐design event was attended by 6 youth, including a youth engager, 5 caregivers including a family engager, and 6 service providers, and was facilitated by researchers with experience working in co‐design processes involving youth. In addition to field notes, researchers wrote memos after conducting interviews about their perspectives on the data being gathered.

**Table 1:** Overview of data sources and collection

Data source	Participants	Timeline of data collection	Type of data	Mode of collection	Output generated
Interview data	Young people aged 16‐24 (n = 19) Caregivers (n = 12) Service providers (n = 14)	August 2015 to April 2016	Qualitative interview data	Semi‐structured interviews conducted face to face lasting ~60 min (ranged from 20 to 90 min). Interviews were conducted three times: at intake to the MyExp study, 6 mo and at 12 mo	Experience maps of touch points across service contact points from each perspective One combined experience map representing and synthesizing touch points from all perspectives
Researcher memos	Research team (n = 3)	August 2015‐August 2017	Qualitative data	Memos written by research team were type‐written and collected	
myEXP application data	Young people (n = 12) Caregivers (n = 6) Service providers (n = 4)	August 2015 to February 2017	Quantitative data Qualitative journal data	Likert scales and open‐ended commentary about experiences of mental health services *my*Comments journal function that allowed free‐form entries by participants	
myEXP co‐design event	Young people (n = 5) Youth engager (n = 1) Caregivers (n = 4) Family engager (n = 1) Service providers (n = 6)	July 21, 2017	Qualitative data augmenting experience maps Co‐designed prototype (drawings and pictures)	Focus groups of like‐participant types (young people, caregivers, service providers) validating experience maps and touch points Prototype development and co‐design with carousel of mixed participant types	Co‐designed prototype

### 2.3 Participant recruitment

Youth were recruited through organizations where they were receiving services and each youth was asked to choose a caregiver to participate, along with the referring service provider^4^ . Service providers represented a range of roles including residential care workers, nurses, mental health counsellors, psychologists and psychiatrists. Service settings included community mental health agencies, hospitals, psychiatric programmes for young people, transition aged services and supportive living. The *my*EXP app questionnaires asked about experiences during arrival into services, the relationship with the provider, information sharing, family involvement, the care plan, feelings upon exiting services, preparedness when moving to a new provider, as well as about the overall experience. These guides were informed by theory and best‐practices cited for coordination of care and transitions from youth to adulthood in mental health services and were the basis for our sensitizing concepts.^26^, ^27^, ^28^ In parallel to the app data, semi‐structured interviews were conducted with youth, caregivers and service providers to ask about experiences at intake to the study, 6 and 12 months.

All data were managed using NVivo software (version 11.0).

### 2.4 Data analysis

#### 2.4.1 Phase 1: Finding and mapping touch points

We used an interpretive phenomenological approach to coding data and identifying touch points, combined with sensitizing concepts from the literature.^29^, ^30^ This approach aligns with the diagnostic stage of EBCD wherein experiences are gathered, and researchers are positioned to record these experiences from an ethnographic stance, consistent with methods aimed at empowering vulnerable groups such as youth.^10^, ^29^ Using the high and low touch points from the data to develop experience maps for each perspective, we descriptively categorized these touch points by type of context, setting, location and type of associated patient quality incident. We first identified the main points along young peoples’ mental health‐care journeys from the three perspectives and then mapped high and low (or positive and negative) touch points during the interface with services (eg, in accessing mental health care, during transfer to and from hospital, during hospital intake, while receiving services, during assessment and treatment, and discharge from hospital. We also noted high and low points that emerged pertaining to caregiver involvement in services and the treatment environment). We refer to these points of interface and other key themes where touch points were identified as “contact points” and take the variation in high and low touch points at these points of interface as indicators of quality variation.

#### 2.4.2 Phase 2: Validation and prototype development

The co‐design process included several rounds of participant engagement, the first of which was a validation exercise of experience maps. As the EBCD process is participatory in nature, we recorded feedback from the validation exercise in real‐time in the like‐participant type focus groups (young people, caregivers, service providers). We then worked with participants to interpret their feedback and support them in developing an overarching question that summarized the problem for the prototype co‐design phase of the event.

During the co‐design work, each group focused on an overarching touch point that captured the core challenge in youth mental health services from their own perspective. The other groups commented in turn on these initial prototypes to enhance them to reflect all perspectives. We focus here on the work of the youth as service users, and the comments of service providers and caregivers to enhance the youth prototype^5^ .

## 3 FINDINGS

### 3.1 Touch points and the continuum of quality mental health care

**Figure 1:**
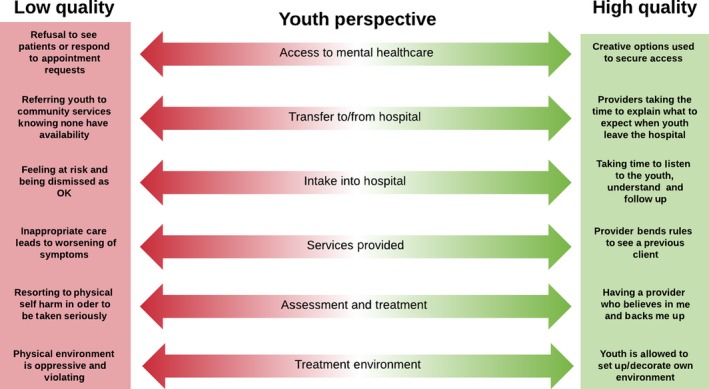
Key themes from young peoples’ perspectives on quality mental health care

**Figure 2:**
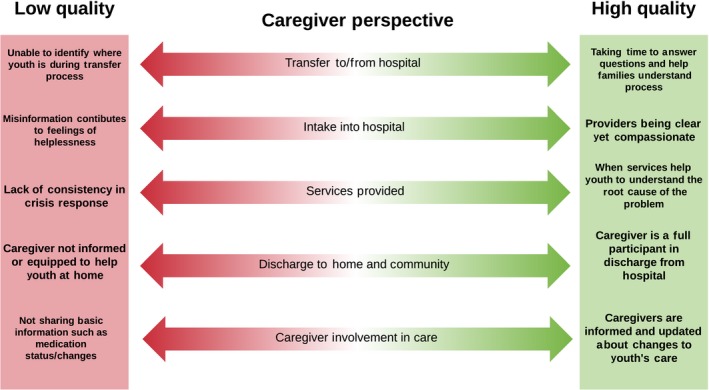
Key themes from caregivers’ perspectives on quality mental health care

**Figure 3:**
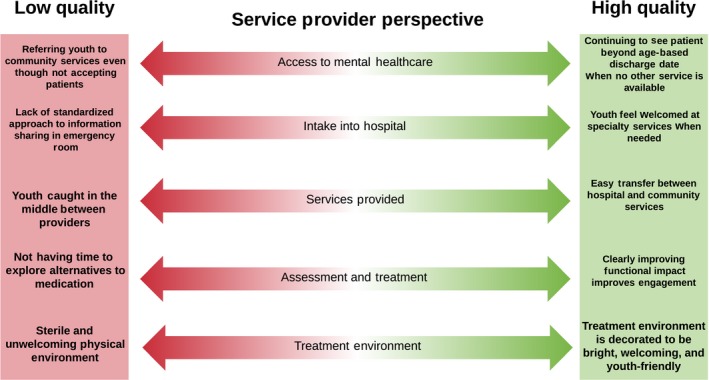
Key themes from service providers’ perspectives on quality mental health care

Figures 1, 2 and 3 present the high and low touch points at each service contact point, for youth, caregivers and service providers, respectively, based on the most salient and frequently reported experiences in the *my*Co‐Design study. In each figure, the high and low ends of the continuum reflect the best and worst dimensions of quality reported by participants from each perspective. By comparing across figures in Table 2, there was considerable overlap in the contact points mentioned by each participant type, but only two contact points where all three groups identified touch points: during hospital intake and in the services provided. Within the common contact points, youth, caregivers and service providers pointed to different highs and lows in their experiences as shown in the three figures. While all of these are important from a quality improvement perspective, we focus here on the youth perspective as service users, and statements from the other perspectives that support the issues raised by youth. As shown in Figure 1, young people described extremes in many areas. Notably, a common influence on their perceived quality of mental health care for youth across the service contact points was the degree to which they felt validated and heard in their interactions with providers.

**Table 2:** Touch points across service contact points and by participant type

Service contact point	Youth	Caregiver	Service provider
Access to mental health care	✓		✓
Transfer to and from hospital		✓	
Intake into hospital	✓	✓	✓
Ongoing care	✓	✓	✓
Assessment and treatment	✓		✓
Treatment environment	✓		✓
Discharge from hospital		✓	
Parent involvement		✓	✓

#### 3.1.1 Access to services

In terms of accessing services, one youth described a high point when the provider explored unique options such as research studies to provide youth with access to the needed services. Service providers also described instances where they have discretion in facilitating access, by bending rules to be able to continue to see youth beyond the typical limits of service provision. For example, one provider described discharging a youth and then accepting the youth as a new referral.

In contrast, another youth who was running out of medication and was very worried about having to go through withdrawal, described not being able to access care from either a paediatrician or from the family physician, and having to seek help at the hospital as a low point. Some acute care hospital‐based providers shared their own frustrations in having to discharge youth to community services, all the while knowing that there were no community services that could accept the youth right away.

The idea is we're supposed to see them for four to six sessions, stabilize, do any kind of medication, monitoring health, teaching, and education. And then we're supposed to refer them to the community services, but of course there are no community services that can pick them up immediately. (Service Provider)

#### 3.1.2 Intake into hospital

For many youth, having providers who listen, understand and follow up was described as a positive touch point, particularly during hospital intake, when they are typically in crisis. However, many youth in our study described arriving at hospital because they were feeling unsafe and yet feeling that staff were dismissive and devalued their experiences during the intake process:

I was never admitted. So, they were just like, “Oh, well, you're okay. You can go home.” And I was just like, “Okay. You're talking to somebody who has scars all up her arm, and tries to commit suicide, and you're going to send me home? (Youth)

Caregivers similarly appreciated being greeted with a compassionate attitude and being given clear directions and explanation of rules during the very stressful experience of presenting their child during hospital intake. This was seen as crucial to feeling positively at an otherwise extremely difficult moment. One caregiver emphasized how important it is to feel good about the people being entrusted with their child's care:

You feel bad enough as a parent leaving your child in the hospital. But when you know they're being left with good staff it makes you feel that much better. (Caregiver)

A community‐based service provider remarked how it was important that the hospital intake services made a youth who was struggling with suicidal ideation feel welcome rather than dismissed, because the youth was trying to be proactive and take responsibility for her mental health. However, service provider participants suggested that such responses are very uneven and depend on the individual intake worker interviewing the youth and the strength of the relationships among providers. One service provider felt that not enough effort is placed on seeing whether a bed is available, with the result that some youth in need are missed, sometimes with disastrous consequences. Another service provider reported that strong collaboration between community services, the psychiatrist and the inpatient unit can enhance continuity of care during transfers across services, including intake to hospital. Otherwise, youth can feel caught in the middle between providers who do not know and trust each other.

#### 3.1.3 Services provided

In terms of youths’ experiences of ongoing service provision, there were many instances where youth described providers who went above and beyond to provide high quality services. For example, one youth who was admitted to an inpatient setting was very appreciative that a nurse who had worked with the youth before went out of her way to be assigned to the youth so as to provide a “familiar face.” However, another youth described being put in the wrong programme in the community, which resulted in a worsening of symptoms.

I was there with the intention of getting help for my depression, and in the process I was given anxiety… I went from being able to walk around a large mall by myself… to getting freaked out in a group of people that I know… in a matter of a couple weeks. (Youth)

#### 3.1.4 Assessment and treatment

Service providers commented that a marked change has occurred over the years in terms of how much time can be spent working with youth, with a resulting reduction in quality.

Yeah, I feel constrained. I feel like I can only do a portion of the work. Not to say it's not helpful, [but] from a theoretical standpoint I feel like it could be so much more helpful, and I just am restricted in what I can do. (Service Provider)

Service providers explained that there is too much reliance on medication during assessment and treatment, because there is not enough time and staff to offer longer term, ongoing care instead. This may contribute to a disconnect between the care youth felt they needed when reaching out for mental health care, and the care they actually received.

#### 3.1.5 Treatment environment

Youth also pointed to the importance of environmental elements in their experiences of quality mental health care. Youth appreciated youth‐friendly environments and communication approaches (such as email or text) as quality enhancing. Youth in residential care were very pleased to be able to decorate their room in a way that suits them.

I actually really like it here because just art on the wall, it's really like you can tell it's like youth orientated and it's focused on us. The people are our age, so they're into our culture. It's nice. (Youth)

However, other environments were described as being highly sterile at best or at worst “traumatizing” and feeling “like a jail.” One youth described feeling violated as a hospital took away all of their belongings and tore apart their clothing to remove anything that could be used for self‐harm. The youth described then being placed in a room that “*someone totally destroyed* … *like punches and kicks everywhere, and there's plaster all over the walls.” (Youth)*

### 3.2 Touch point for co‐design

Following the synthesis of touch points focusing on high and low quality mental health care for youth, each perspective had an overarching touch point that resonated most with their experiences. The touch point that resonated most for youth, and which was validated by youth and selected for co‐design, was that *poor communication and collaboration can impede quality care*. The following components of communication and collaboration across services that strongly influenced the quality of CYMHS were identified in the youth experience data:

- Youth often lose access to their network of trusted providers during transitions between services.
- Youth and parents feel vulnerable and like they don't know where to turn when different providers adopt inconsistent treatment approaches.
- Youth are frustrated that they have to retell their stories over and over because of poor communication among providers and services.
- Communication works well within a provider's trusted network of colleagues. Frustrations arise when communicating with providers outside that network.

After developing a problem statement related to their overarching touch point, young people focused on their interactions with service providers as a central component of mental health care that could benefit from a prototyped design solution. The priority question for youth was “How do we create an environment where you feel respected, and you feel like what you say matters?” Youth elaborated that such an environment is where:

- Providers see my strengths in the situation, and I am not being judged based on the past;
- Environmental factors make me feel comforted and reduce anxiety;
- I am taken seriously, especially at the hospital when I do not want to be there, instead of having providers say that I am ‘looking for attention.’

**Figure 4:**
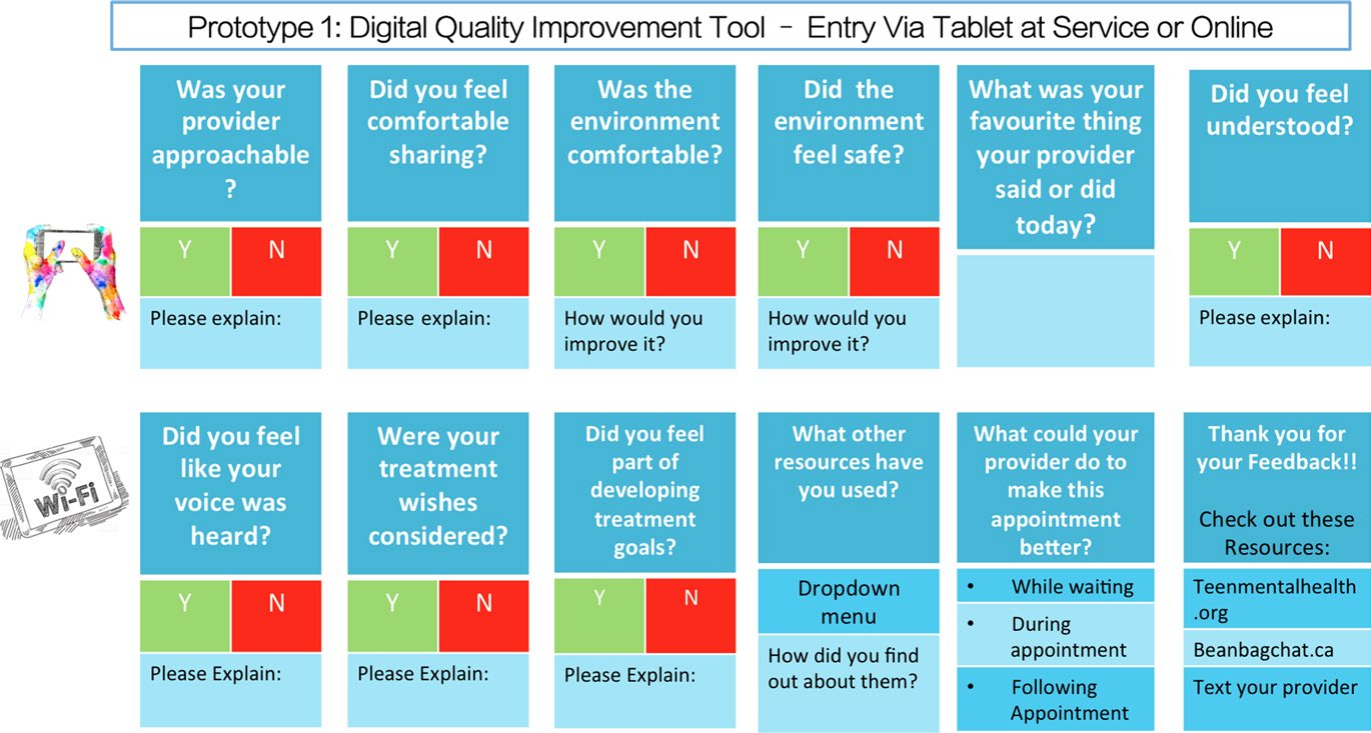
Youth prototype

Participants described feeling vulnerable when being critical of providers and discussed the need for a way to communicate more comfortably with providers who might mean well, but not realize they were saying or doing harmful things. One youth suggested the need for an anonymous feedback tool and the group landed on a web‐based tool available on a tablet in the waiting room or online at another setting to record their care experiences as a solution. Such a tool could provide the provider and organization with information across key elements of service delivery and design and could contribute to ongoing quality improvement based on user feedback. It could also be used in a cross‐organizational context for youth‐centred quality improvement. The youth collectively developed a prototype that consisted of online questions that youth could answer on a tablet following each provider visit. Figure 4 presents the questions that youth proposed for their prototype. Young people stressed the importance of having the feedback be anonymous but provider‐specific, so that providers could know what they could improve or do to be more responsive to youths’ treatment needs.

Caregivers and service providers were highly supportive of the prototype developed by the youth and recognized this as a way to give voice to young people. Caregivers were interested in whether such a tool would be available for them to provide their own feedback. Service providers had questions about where the data would be held and by whom, and how to ensure organizations and individual service providers would use the feedback to improve service delivery. Some service providers suggested that aggregate responses to these questions could be used for ongoing quality improvement purposes.

## 4 DISCUSSION

Our findings suggest that the EBCD process was helpful in engaging participants’ voices and perspectives, about their experiences of mental health‐care quality. Youths’ experiences pointed to the importance of having access to services, feeling heard, validated and responded to, and having service providers who could adapt to their individual needs. The co‐design event allowed youth to validate the relative importance of communication and collaboration to receiving high quality care, and to work together with other youth, as well as caregivers and service providers, to co‐design ways to provide feedback to service providers about the quality of their interactions. Overwhelmingly, youth and caregivers identified the importance of service provider responsiveness to youth as key to delivering high quality care within a complex and fragmented system. Despite being highly motivated to improve mental health services for young people, service providers struggled with communication and information‐sharing across organizations arising from system fragmentation. This structural challenge reflects not only the Canadian and Ontario context as discussed above, but has also been identified as an issue for CYMHS in many settings.^31^, ^32^, ^33^ Improvements to communication and information sharing are recognized as being critical to improving efficiency of systems, coordinated care, and better health and mental health outcomes.^34^, ^35^

The range of high and low touch points shown in Figures 1, 2 and 3 could serve as a framework and starting point to develop a full range of mental health quality indicators across the system of care by stakeholder perspective. While there are some common areas across perspectives, there are also distinct differences. For example, access is an issue for both youth and service provider participants; however, there are different nuances in interpretation. For youth, high quality was reported when providers went out of their way to obtain access through initiatives like enrolling youth in research studies, which corresponds to their emphasis on the importance of the individual provider to service quality. In contrast, service providers pointed to organizational and system factors as affecting their ability to provide quality services, such as having to discharge patients to community services that they know are not available, or having to bend rules to keep youth beyond a discharge date that is required by organizational or system level policies. It was disconcerting how often youth and family participants pointed to providers who “bent the rules” in order to meet their needs as indicators of service quality. In a truly quality service, rules would support quality and not need to be bent to deliver quality.

Two particular hotspots from all perspectives were having care available in the community to avoid the need for hospitalization where possible and that the concerns of youth and families should be taken seriously in order to avoid the risk of serious physical and psychological harm. We were alarmed at the frequency with which we heard that being dismissive of a youth's concerns at hospital intake could result in serious self‐harm or suicide attempt upon return to the home or community, where system supports are not available. This repeated theme of youth perceiving that their needs were dismissed is clearly aligned with their selected solution: an anonymous feedback tool by which youth voices and experiences can be heard by individual service providers and systems as whole in order to improve the quality of youth mental health services.

### 4.1 Implications for policy and practice

When it comes to improving quality, the experiences reported here suggest that there is a need to devise ways to overcome structural barriers to improve service coordination at the organizational and system levels.^36^ It is clear that the efforts of some providers to go above and beyond and advocate for high quality services for youth is recognized and appreciated by young people and families. The “wicked” policy problem of improving youth mental health care spans many organizations across multiple systems and sectors. When facing this problem, service providers play an essential role in engaging and enabling youth, caregivers and service providers to work together to facilitate ongoing quality improvement processes.^36^, ^37^, ^38^, ^39^ Many youth and families pointed to experiences of high quality care related to efforts made by particular providers. This suggests there is room to improve quality through provider training in the short to medium term, while addressing systemic and organizational barriers over the medium to longer term^6^ . In addition, considerable opportunity exists to improve quality by making service environments and approaches more youth‐friendly and by empowering and communicating with youth in ways that are more accessible to youth, including using text and email.

In order to develop a higher quality system, the various perspectives need to be considered. In the EBCD process, all perspectives are heard and each group is involved in developing prototypes that reflect their various needs. Youth in our study developed a prototype of a short, simple electronic feedback tool targeting domains of importance to youth that could be used to build quality improvement into mental health service delivery on an ongoing basis, particularly with respect to the provider factors they identified. The feedback tool serves as an important point of departure for ongoing quality improvement in CYMHS both within individual services and across networks of providers as it showcases how important provider communication and collaboration are in young people's journeys through mental health care. Service provider and caregiver participants in our co‐design process supported and augmented this prototype with feedback on how this could work in practice, and how the data generated could be used within service delivery organizations. We anticipate that this process can ensure that organizations and providers develop the mutual understanding and respect for each others’ perspectives that can facilitate openness in receiving and integrating such constructive criticism—a condition necessary for quality improvement according to the literature.^40^ As is the case for any prototype, is expected to need further refinement, but may be a useful starting point that services in Canada and elsewhere can adopt as a way to enhance system quality going forward.

### 4.2 Limitations

Each local health‐care system is unique, and findings from this study may not be immediately transferable to other settings. We do not claim that the experiences of participants of this study represent larger groups of service users. To our knowledge, this is the first EBCD study to explore how experiences can inform youth‐driven quality improvement practices in CYMHS; however, we did find analogous findings in many cases to studies examining patient engagement, experiences and young people's mental health.^7^, ^41^, ^42^, ^43^ The choice to develop a feedback tool as a way to improve quality and service experiences from the youth perspective emerged organically from the EBCD process and was not an explicit objective of the study.

## 5 CONCLUSION

A clear message across all participant types is that that the time has come to listen to and respect youth and family experiences. A high quality mental health system can no longer be seen as one that dismisses young people and families in crisis only to return home unsupported and ill‐informed when at serious risk of self‐harm and suicide. The EBCD process can help to foster mutual respect and understanding and break down the attitudinal barriers that inhibit collaboration across users and other key stakeholders in improving service design, experiences and ultimately mental health and well‐being outcomes. The prototype presented here is a starting point for such engagement. By using EBCD to capture in‐depth data regarding experiences of young people, their caregivers and service providers, study participants have begun to establish a baseline for acceptable quality of mental health care for young people.

## CONFLICT OF INTEREST

None declared.

## Notes

1 In an effort to improve local coordination, Ontario has partially regionalized its health‐care system, by establishing fourteen Local Health Integration Networks (LHINs) covering the geographic expanse of the province. LHINs are responsible for planning and funding health services within their boundaries, with funding from capital budgets provided by the Ontario Ministry of Health and Long‐term Care (MoHLTC). However, community‐based child and youth mental health services are under the purview of the Ontario Ministry of Child and Youth Services, which has a separate set of lead agencies, with different geographic boundaries than those of the LHINs. This complicates transitions between children's and adult mental health services, care coordination across agencies and with mental health care in secondary and post–secondary education, addictions services and the justice system.

2 Young people entered their appointment times into the app, and after these appointments, the smartphone app sends online questionnaires to the caregiver and service provider. Caregivers and service providers also answered questions about their experiences throughout the young person's journey. The MyExp app commentaries were also used in the analyses.

3 The daylong *my*Co‐Design event was held in July 2017 and included all three participant types from the duration of the study. Participants were contacted in April 2017 and invited to participate in the co‐design process. Five young people, four caregivers and six service providers attended, as well as one parent engager and one youth engager who had participated in other EBCD events led by the PI GM. Further information about the design and outcomes of the event will be found in Mulvale et al. (forthcoming).

4 Note that for some youth the service provider was also selected as the informal support rather than a caregiver.

5 Service providers and caregivers developed prototypes that were designed to improve experiences but did not take the form of a quality feedback tool and so are not reported here, but are discussed in forthcoming publications.

6 Prototypes to address these structural barriers were also developed and will be discussed in forthcoming work.
